# Controlled release herbicide formulation for effective weed control efficacy

**DOI:** 10.1038/s41598-024-53820-8

**Published:** 2024-02-20

**Authors:** Santosh Kumar Paul, Yunfei Xi, Peter Sanderson, Ravi Naidu

**Affiliations:** 1 Global Centre for Environmental Remediation (GCER), The University of Newcastle, ATC Building, Callaghan, NSW 2308 Australia; 2 CRC for Contamination Assessment and Remediation of the Environment (CRC CARE), The University of Newcastle, ATC Building, Callaghan, NSW 2308 Australia; 3 Agronomy Division, Bangladesh Agricultural Research Institute (BARI), Joydebpur, Gazipur 1701 Bangladesh; 4 Central Analytical Research Facility (CARF) & School of Chemistry and Physics - Faculty of Science, Queensland University of Technology, Brisbane, QLD 4001 Australia

**Keywords:** Herbicide, Kinetics, Thermodynamics, CRFs, Weeds, Environmental sciences, Materials science

## Abstract

Controlled release formulation (CRF) of herbicide is an effective weed management technique with less eco-toxicity than other available commercial formulations. To maximise the effectiveness of CRFs however, it is crucial to understand the herbicide-releasing behaviour at play, which predominately depends on the interaction mechanisms between active ingredients and carrier materials during adsorption. In this study, we investigated and modelled the adsorption characteristics of model herbicide 2,4-D onto two organo-montmorillonites (octadecylamine- and aminopropyltriethoxysilane-modified) to synthesise polymer-based CRFs. Herbicide-releasing behaviour of the synthesised CRF microbeads was then analysed under various experimental conditions, and weed control efficacy determined under glasshouse conditions. Results revealed that adsorption of 2,4-D onto both organo-montmorillonites follows the pseudo-second-order kinetics model and is predominately controlled by the chemisorption process. However, multi-step mechanisms were detected in the adsorption on both organoclays, hence intra-particle diffusion is not the sole rate-limiting step for the adsorption process. Both organoclays followed the Elovich model, suggesting they have energetically heterogeneous surfaces. Herbicide-releasing behaviours of synthesised beads were investigated at various pH temperatures and ionic strengths under laboratory and glasshouse conditions. Furthermore, weed control efficacy of synthesised beads were investigated using pot studies under glasshouse condition. Desorption studies revealed that both synthesised microbeads have slow releasing behaviour at a wide range of pHs (5–9), temperatures (25–45 °C), and ionic strengths. The results also revealed that synthesised microbeads have excellent weed control efficacy on different broad-leaf weed species under glasshouse conditions.

## Introduction

In global agricultural markets, weeds are estimated to be the highest cause of potential yield loss (34%) when compared with animals (18%) and pathogens (16%)^1–4^. Weeds adversely affect crop production in terms of quality, economy and environment^4–6^. Both narrow and broad-leaf weeds have detrimental impacts on crop fields, however broad-leaf weeds are considered the biggest weed pest worldwide^1,7,8^. Some invasive broad-leaf weeds release toxicants (Allelochemicals) through their roots, impacting crop growth and production^4,7,8^. Therefore, effective weed management is vital for retaining desired crop quality and production rates^4,8–10^.

Herbicides have long been the favoured weed management practice among farmers, above all other physical, cultural, mechanical and biological methods, due to their cost-effectiveness, fast-acting and high weed control efficiency and their easy application^2,3,11,12^. However, most of the available herbicide formulations cannot control the release of the herbicide’s active ingredients (AIs), and their burst release immediately after application can cause severe eco-toxicity in the soil and nearby water bodies. When these persistent pollutants reach drinking water, they are known to pose serious human health risks^6,13,14^. Moreover, weed resistance to herbicides due to indiscriminate application has become a major concern in modern agriculture worldwide^2,15^. Without a changed approach, this problem will only grow.

When controlled release formulations (CRFs) of herbicides are used, the AIs that have been loaded into/onto the carrier materials are slowly released into the soil instead of burst releasing after application, minimising their movement through soils and ground water^16–18^. CRFs could therefore be an effective alternative to traditional herbicides, reducing the likelihood of ecotoxicity and increased herbicide resistance.

Effective CRFs are primarily dependent on the physicochemical properties of the carrier materials and adsorption conditions during herbicide loading onto the carriers^6,11,12,15,18,19^. Initial rapid adsorption takes place on the easily accessible external sites of the carrier (adsorbents), resulting in fast release during desorption. Conversely, the sorption process becomes slower on the less accessible internal sites. This acts as a rate-determining step for the whole adsorption process and contributes to overall slow-releasing properties^12,20–22^. Therefore, it is important to understand the sorption kinetics in order to synthesise an effective formulation. Nano-structured clay minerals were chosen in this study over other potential carrier materials due to their natural abundance, low cost, eco-friendliness, non-toxicity, easy availability, large surface area, biodegradability and biocompatibility^6,11^.

The purpose of this study was to investigate the sorption pattern of a model herbicide (2,4-D) onto octadecylamine (ODA) and aminopropyltriethoxysilane (APTES) modified organo-montmorillonites to synthesise polymer-based CRFs, along with herbicide desorption behaviour of the synthesised microbeads at various pH (acidic, neutral, and alkaline), temperatures (20–45 °C) and ionic strengths. A non-toxic, biodegradable natural polymer “sodium alginate” were used to synthesis 2,4-D loaded polymeric beads for CRFs. This paper will outline the herbicide sorption kinetics and thermodynamics along with probable interaction mechanisms between carrier materials and 2,4-D during adsorption. Lastly, the weed control efficacy of the synthesised CRF microbeads was investigated under glasshouse conditions using broad-leaves weed species. Suggestions are made for devising eco-friendly, sustainable and highly effective CRFs of anionic herbicides that will have the desired release properties.

## Experimental sections

### Materials and reagents

Two organoclays (ODA and ODA + APTES-modified montmorillonites) were purchased from Sigma-Aldrich, Australia and denoted as MMT1 and MMT2, respectively. These organoclays were used without further purification; some of their properties, provided by the supplier, have been presented in Supplementary Table 1. The model herbicide 2,4-D (PESTANAL®, analytical standard, purity > 98%) was also purchased from Sigma-Aldrich, Australia and its physicochemical properties are also presented in Supplementary Table 2. Sodium alginate and calcium chloride (CaCl_2_) were used for microbead synthesis, where analytical grade hydrochloric acid (HCl, 37%) and sodium hydroxide (NaOH, > 99% purity) were used for pH adjusting and all of those were purchased from Sigma-Aldrich, Australia. The seeds of two broad-leaf weed species, Fleabane (*Conyza bonariensis*) and Sowthistle (*Sonchus oleraceus*), both members of the daisy (Asteraceae) family, were collected from Weed Research Group, Department of Agriculture and Fisheries, Leslie Research Facility, Toowoomba, Queensland. Seeds of another broad-leaf weed species that commonly grows in wheat, maize and sugarcane fields, Lamb’s Quarters or Pigweed (*Chenopodium album*), from the amaranth (Amaranthaceae) family, were collected from a home garden at Mount Hutton, New South Wales. A commercial herbicide formulation (2,4-D Amine 300, AI 300 gL^−1^) was purchased from Conquest Crop Protection Pty Ltd., Osborne Park, Western Australia.

### Herbicide sorption kinetics

Batch adsorption experiments were carried out following the methodology stated in the supplementary material. Our previous investigation revealed that adsorption of 2,4-D onto both organoclays was highest under acidic conditions (pH.3). Therefore, adsorption kinetics studies were investigated at pH.3. Five sorption kinetics models—pseudo-first-order (PFO), pseudo-second-order (PSO), Elovich, intra-particle diffusion and two-constant rate models—are described in the supplementary sections and were applied to the experimental data in order to determine the mechanisms of sorption behaviour.

### Soil sampling and characterisation

Experimental soils were collected from Fletcher, NSW, Australia (latitude 32° 52′ 36′′ S and longitude 151° 38′ 22′′ E). Physicochemical properties of the collected soils were investigated following methods stated in the supplementary sections, and results are presented (Table 1).

**Table 1:** Physicochemical properties of selected soil sample.

Parameters	Properties	Parameters	Properties
Textural class	Sandy loam	Fe (%)	0.03
SA (m^2^g^−1^)	1.87	Ca (%)	0.71
Sand (%)	63.80	Na (%)	0.08
Silt (%)	23.80	K (%)	0.03
Clay (%)	12.40	Mg (%)	0.16
EC (µs cm^−1^)	190.5	N (%)	0.41
CEC (meq 100 g^−1^)	9.70	S (%)	0.08
pH (in MQ-water)	6.60	Major mineral compound	Quartz, Albite, Oligoclase, Sodalite
TOC (%)	1.29		
Al (%)	0.01		

### Microbeads synthesis

Herbicide AIs were loaded onto the organoclays at pH.3 with a solution concentration of 700 mgL^−1^ to confirm maximum adsorption^11,12^. Organoclays (0.25% w/v) were added into the herbicide solution then shaken continuously for 2 h. The suspensions were centrifuged at 4500 rpm (model; Sorvall LYNX 4000 Centrifuge, Thermo Scientific, Germany) for 15 min at 25 °C. Supernatants were then filtered and the herbicide-loaded organoclays were dried in an oven at 60 °C for 24 h, then finely powdered using a mortar and pestle.

The 2,4-D adsorbed organoclays were added to a homogenous sodium alginate solution (distilled water and sodium alginate @3% w/v) and stirred (150 rpm) using a magnetic stirrer for 24 h, while at room temperature^23,24^. The 2,4-D/organoclay-alginate suspension was then taken into a 10 ml syringe and extruded dropwise into a cross-linking solution (3% w/v CaCl_2_ solution) using a needle with an internal diameter of 0.45 mm. The 2,4-D/organoclay-alginate beads were instantaneously formed in the cross-linking solution and left for an additional 4 h under continuous stirring to ensure complete gelling. The beads were rinsed with distilled water, freeze-dried and kept in glass bottles. The microbeads were synthesised separately using MMT1 and MMT2 as the organoclay carriers and denoted as MMT1_Beads_ and MMT2_Beads,_ respectively.

### Desorption studies

50 mg of MMT1_Beads_ and MMT2_Beads_ were each placed into a different 50 ml centrifuge tube with 10 ml pre-pH-adjusted (pH.5) MQ-water, then shaken (50 rpm) at 25 °C. The water was separated from the beads using a 0.25 µm CA-filter and replaced by 10 ml pre-pH-adjusted (pH.5) MQ-water. The process was continued until a considerable amount of 2,4-D was detected in the water using a UV–Vis spectrophotometer. The experiment was repeated using pre-pH-adjusted MQ-water at pH 7 and 9. Similar investigations were then carried out at different temperatures (25 °C, 35 °C and 45 °C), ionic strengths (0.01 M NaCl and 0.03 M NaCl) and index cation (0.01 M CaCl_2_). All studies were replicated twice, and the mean values reported.

A replicated pot experiment was conducted in a glasshouse to investigate herbicide desorption behaviour in normal soil conditions. Prior to this experiment, maximum water holding capacity (WHC) of the soil (380 ml kg^−1^) was calculated, and 70% WHC was maintained throughout the experiment. Air-dried soil samples (1 kg per pot) were placed in each pot (118 $\times$ 125 mm) and the calculated amounts of water were added to reach the desired experimental scenario. Pots were watered regularly with limited drainage to maintain 70% WHC. Synthesised microbeads (1000 mg pot^−1^) were put into the soil (5 cm depth). A soil-moisture sampler (Rhizosphere Research Products, Rhizon, MOM model, Wageningen, The Netherlands) with mean pore size of 0.15 μm was used to collect soil pore water (SPW)^25^, inserted near the beads into the soil and placed at a 45° angle to the horizon. Only one SPW sample (~ 10 ml) was collected from each pot, at one of a number of pre-selected time intervals throughout the experiment, then SPW samples were analysed to detect 2,4-D.

### Weed control efficacy

A replicated pot experiment was conducted to investigate weed control efficacy of synthesised microbeads. Experimental pots containing air-dried soil samples (1 kg pot^−1^) were placed in the glasshouse. The pots were slowly watered with tap-water (380 ml pot^−1^) using a spraying can and left for two days for field conditions. The soils were pulverised with a small stick and weed seeds were sown into the pots. Pots were watered regularly with limited drainage to maintain 70% WHC for better seed germination. After germination, weed seedlings were thinned out on the 20th and 30th days after sowing (DAS) to keep the desired number of plants per pot for better growth and development. Sowthistle grows faster than Fleabane followed by Pigweed. Therefore, the synthesised microbeads and commercial herbicides were applied separately at 40 DAS, 50 DAS and 55 DAS for Sowthistle, Fleabane and Pigweed, respectively. An untreated fresh pot for each weed species served as the control treatment. The number of weeds per pot was counted before herbicide application. The weed control efficacy of the synthesised microbeads was calculated in comparison with control treatment. Whole experiments were replicated twice, and mean values presented.

$${\text{Weed}}\;{\text{Control}}\;{\text{Efficiency}}\;\left( {{\text{WEC}}} \right) = \frac{{{\text{Dry}}\;{\text{wt}}.\;{\text{of}}\;{\text{control}}\;{\text{plot}} - {\text{Dry}}\;{\text{wt}}.\;{\text{of}}\;{\text{specific}}\;{\text{plot }}}}{{{\text{Dry}}\;{\text{wt}}.\;{\text{of}}\;{\text{control}}\;{\text{plot}}}} \times 100$$

## Results and discussions

### Various sorption kinetics models

The kinetic studies revealed that both organoclays have strong affinity to 2,4-D. Adsorption was fast initially and reached equilibrium within 20 min, however experiments were continued for 120 min to ensure equilibration. In order to investigate the 2,4-D sorption behaviour with each organoclay the experimental data were fitted to the five kinetic models. Correlation coefficient (R^2^) values were then calculated for each model to establish the suitability of each to describe the sorption kinetics of 2,4-D with the organoclays. Results are discussed in the following sections.

#### Pseudo-first-order kinetics model

**Figure 1:**
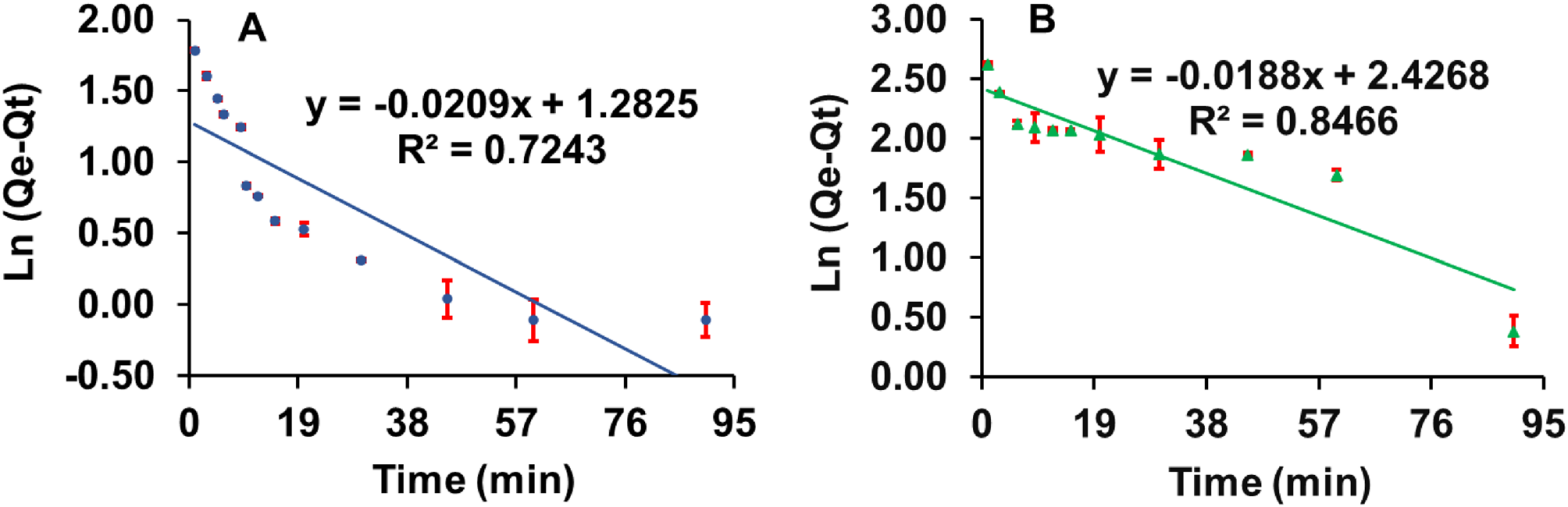
Pseudo-first-order kinetic model for 2,4-D adsorption onto the organo-montmorillonites; (**A**) MMT1, (**B**) MMT2.

Lagergren’s pseudo-first-order (PFO) rate constant (g mg^−1^ min^−1^) for MMT1 and MMT2 were 0.02 and 0.01 respectively (Fig. 1). The calculated equilibrium adsorption capacity, Q_e-cal_ (mg g^−1^) for MMT1 and MMT2 were 3.61 and 11.32, respectively, which were much lower than the experimental equilibrium sorption capacity, Q_e-exp_ (mg g^−1^) (Table 2). The low R^2^ values (Table 2) suggest that PFO is not a suitable kinetics model to describe the experimental adsorption process^26,27^.

**Table 2:** Parameters of various kinetic models.

Organoclays	Q_e-exp_ (mg g^−1^)	Pseudo-First-Order			Pseudo-Second-Order			Elovich		
		Q_e-cal_ (mg g^−1^)	K_1_ (g mg^−1^ min^−1^)	R^2^	Q_e-cal_ (mg g^−1^)	K_2_ (g mg^−1^ min^−1^)	R^2^	α (mg g^−1^ min^−1^)	β (g mg^−1^)	R^2^
MMT1	67.16	3.61	0.02	0.7243	67.11	0.04	0.9990	1.97 X 10^21^	0.79	0.9354
MMT2	52.76	11.32	0.01	0.8466	52.36	0.01	0.9967	1.91 X 10^7^	0.41	0.8878

#### Pseudo-second-order kinetics model

**Figure 2:**
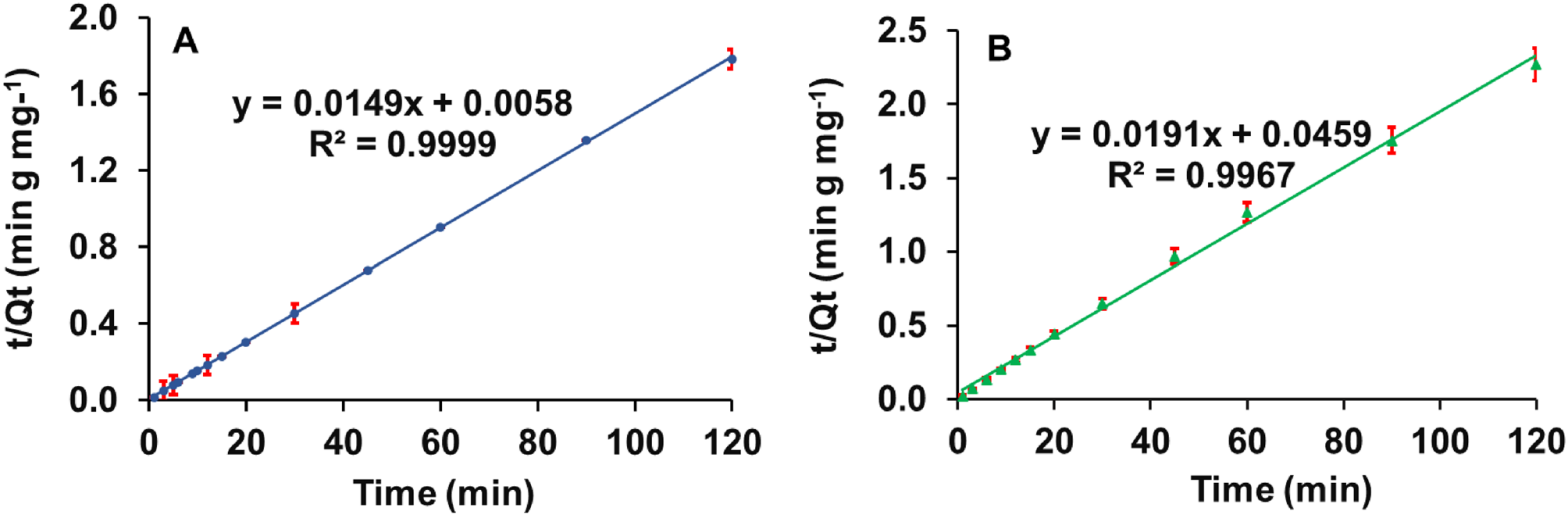
Pseudo-second-order kinetic model for 2,4-D adsorption onto the organo-montmorillonites; (**A**) MMT1, (**B**) MMT2.

The pseudo-second-order (PSO) kinetic model can be expressed in a mathematical equations, and the model is well fitted to the experimental data and with based on the correlation between the fit of the model to data, the R^2^ values were obtained (Fig. 2). The R^2^ values of the PSO kinetics model for both organoclays were significantly higher than for PFO (Table 2). Moreover, the calculated equilibrium adsorption capacity, Q_e-cal_ (mg g^−1^) for MMT1 and MMT2 aligns well with the experimental equilibrium sorption capacity, Q_e-exp_ (mg g^−1^) (Table 2). Therefore, it can be assumed that the adsorption of 2,4-D to the experimental organoclays follows the PSO kinetic model rather than the PFO model, hence chemisorption is the rate-limiting step of the adsorption process^28–31^.

It must be noted however that adsorption of solutes to sorbents is controlled by several factors. Along with physicochemical properties of the solutes (adsorbates) and sorbents (adsorbents), adsorption conditions greatly influence the rate of adsorption^26,32^. Hence it can sometimes be difficult to interpret the results in respect to various adsorption kinetics models because the sorption rate for the same solute and sorbents may be well fitted to different kinetics models under different adsorption conditions. For instance, PSO kinetic models were suitably fitted and well described at lower initial concentrations while the PFO kinetic model was well fitted at a higher initial concentration of the solute (adsorbates)^27,33^. Generally, the unoccupied adsorptive sites and concentration of solutes are constantly decreasing with adsorption times, so PSO is better fitted than PFO in the later stage due to the decreasing concentration of herbicide AIs and reduced number of unoccupied adsorptive sites^28,34,35^. In reality, the adsorption process is a very complicated and continuous phenomenon, making it sometimes difficult to establish which process predominates and which kinetic model fits best to the adsorption process. However, higher R^2^ values are generally the best indicator of well-fitting kinetic models used to explain the adsorption process.

#### Elovich model

**Figure 3:**
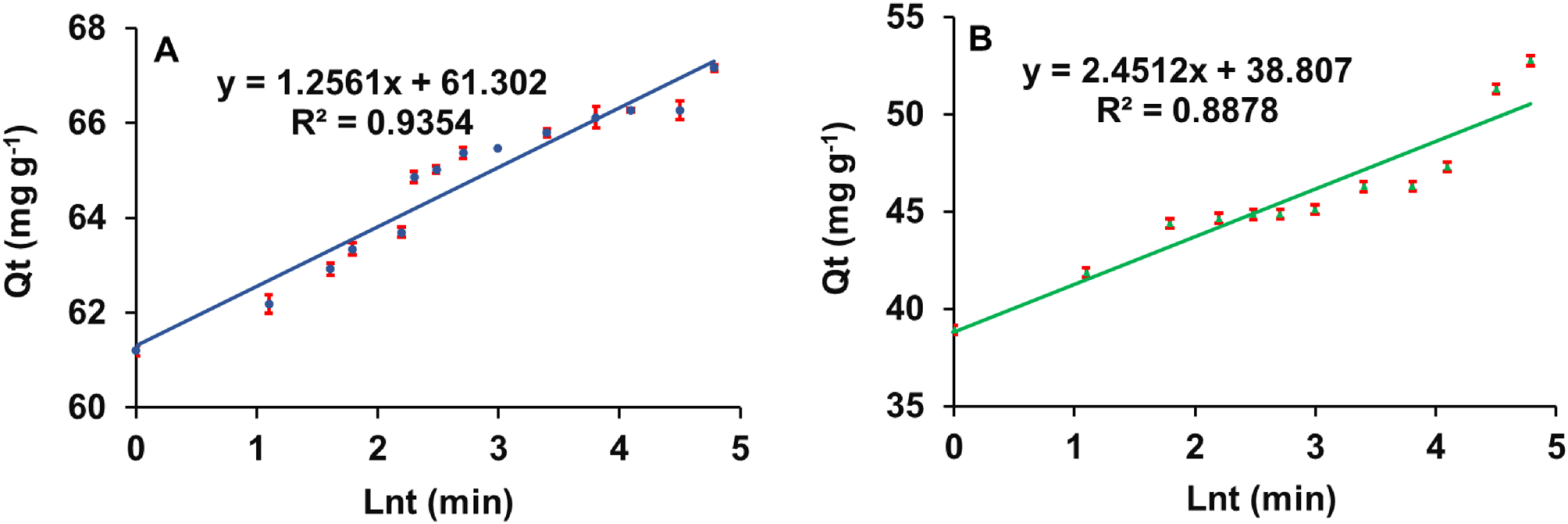
Elovich model for 2,4-D adsorption onto the organo-montmorillonites; (**A**) MMT1, (**B**) MMT2.

The Elovch model was well fitted to experimental data, and results revealed that both organoclays follow the Elovich model, suggesting the organoclays have an energetically heterogeneous surface (Table 2). The intercept values of the plot for MMT1 (61.30) and MMT2 (38.80) are considered to be the initial adsorbed amount (mg g^−1^) of 2,4-D at rapid adsorption phase (Fig. 3). These results suggest that maximum (> 90% for MMT1 and > 70% for MMT2 of total adsorption at equilibrium) amounts of adsorption took place at the initial rapid adsorption phase, which might be attributed to a hydrophobic interaction between 2,4-D and organoclays.

#### Intra-particle diffusion model

**Figure 4:**
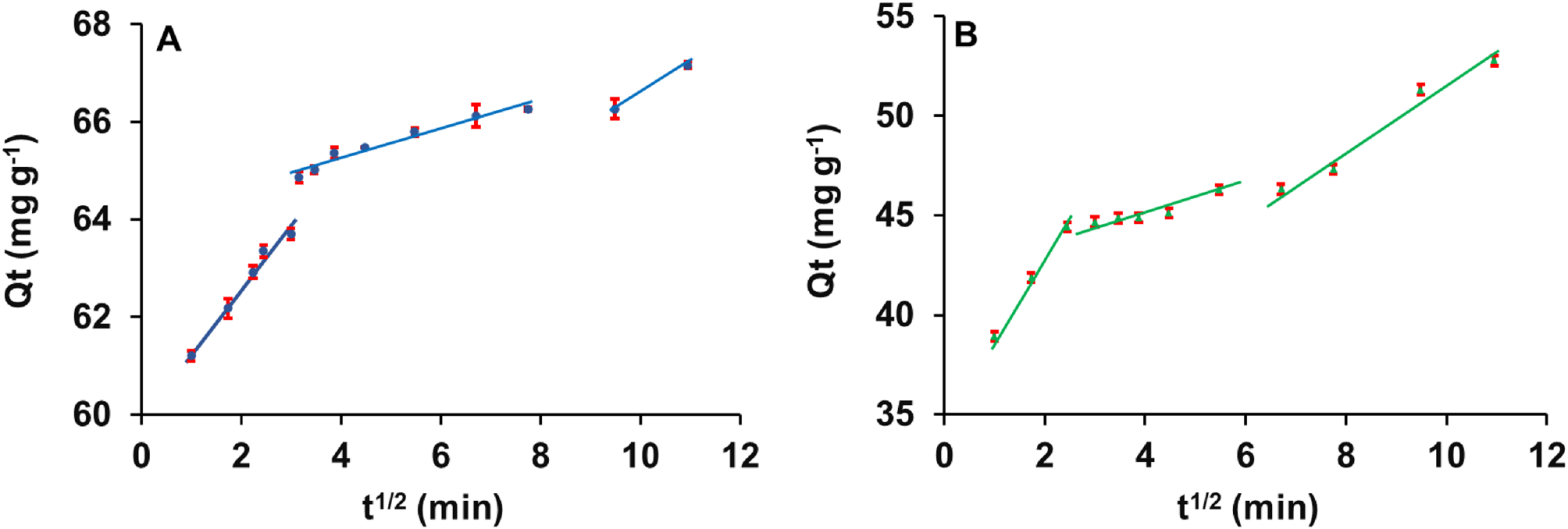
Intra-particle diffusion model for 2,4-D adsorption onto the organo-montmorillonites; (**A**) MMT1, (**B**) MMT2.

The model was fitted to the experimental data and parameters were calculated from the slope and intercept of the plot Q_t_ versus t^1/2^ (Fig. 4). The results show that intra-particle diffusion is not the sole rate-limiting step for the entire adsorption process since the plot did not pass through the origin. The non-linearity of the plots indicates multiple rate-limiting steps were involved throughout the adsorption process for both organoclays. Therefore, it could be assumed that adsorption of the herbicide onto the organoclays involves a multi-step adsorption process: (i) rapid surface adsorption or external diffusion; (ii) intra-particle diffusion to mesopores; and (iii) diffusion to the micro- and macropores.

The mass transfer of solute from aqueous phase (solution) to solid phase (sorbents) is a complex phenomenon and single and/or multi-step adsorption processes could be involved, including surface-diffusion, film-diffusion, intra-particle diffusion, pore-diffusion, and pore-adsorption^36–38^. Adsorption of solutes to the porous materials is significantly influenced by the porosity of the adsorbents, with mesopores being the faster adsorptive sites of the adsorbents^32^. In the multi-linear adsorption process, it can be assumed that the second and third slower adsorption steps can be attributed to intra-particle diffusion through micropores and macropores and these are therefore rate-limiting steps of the entire adsorption process^39^.

#### Two-constant rate model

**Figure 5:**
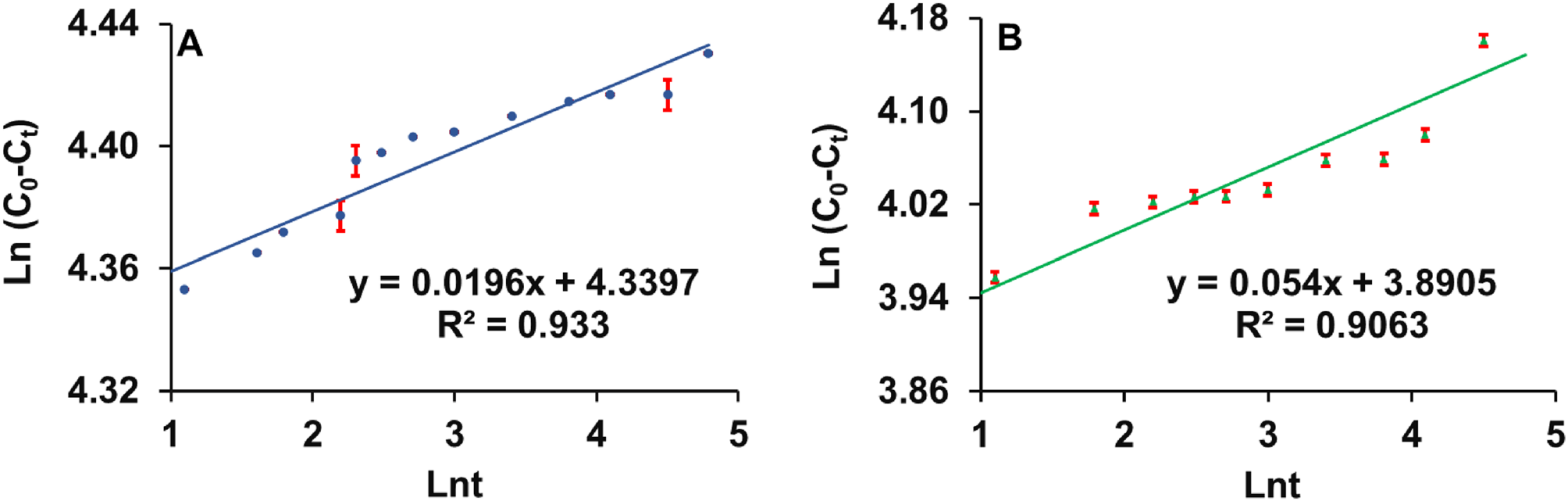
Two-constant rate model for 2,4-D adsorption onto the organo-montmorillonites; (**A**) MMT1, (**B**) MMT2.

The higher R^2^ values (0.9330 and 0.9063 for MMT1 and MMT2, respectively) indicate a positive correlation with the experimental data suggesting chemisorption (Fig. 5 and Table 3)^20,40^. The chemisorption can be attributed to strong interactions between adsorbents and adsorbates resulting from the exchange of valence electrons between them (adsorbents and adsorbates) through the chemical process^40^. This strongly supports the heterogenous surface of both organoclays.

**Table 3:** Adsorption kinetic parameters of Intra-particle diffusion and Two-constant rate model.

Organoclays	Intra-particle diffusion model									Two-constant rate model			
	Diffusion rate constant (mg g^−1^ min^−1/2^)			Intercept (mg g^−1^)			Regression coefficient			Rate constant a (mg g^−1^ min^−1^)	Rate coefficient b	Regression coefficient R^2^	
	K_p1_	K_p2_	K_p3_	C_1_	C_2_	C_3_	R_1_^2^	R_2_^2^	R_3_^2^				
MMT1	1.13	0.30	0.99	59.96	64.06	1.56	0.9810	0.9529	**1.0000**	4.34	0.02	0.9330	
MMT2	3.79	0.51	1.72	35.19	43.05	34.28	0.9985	0.8944	**0.9558**	3.89	0.05	0.9063	

### Desorption studies

**Figure 6:**
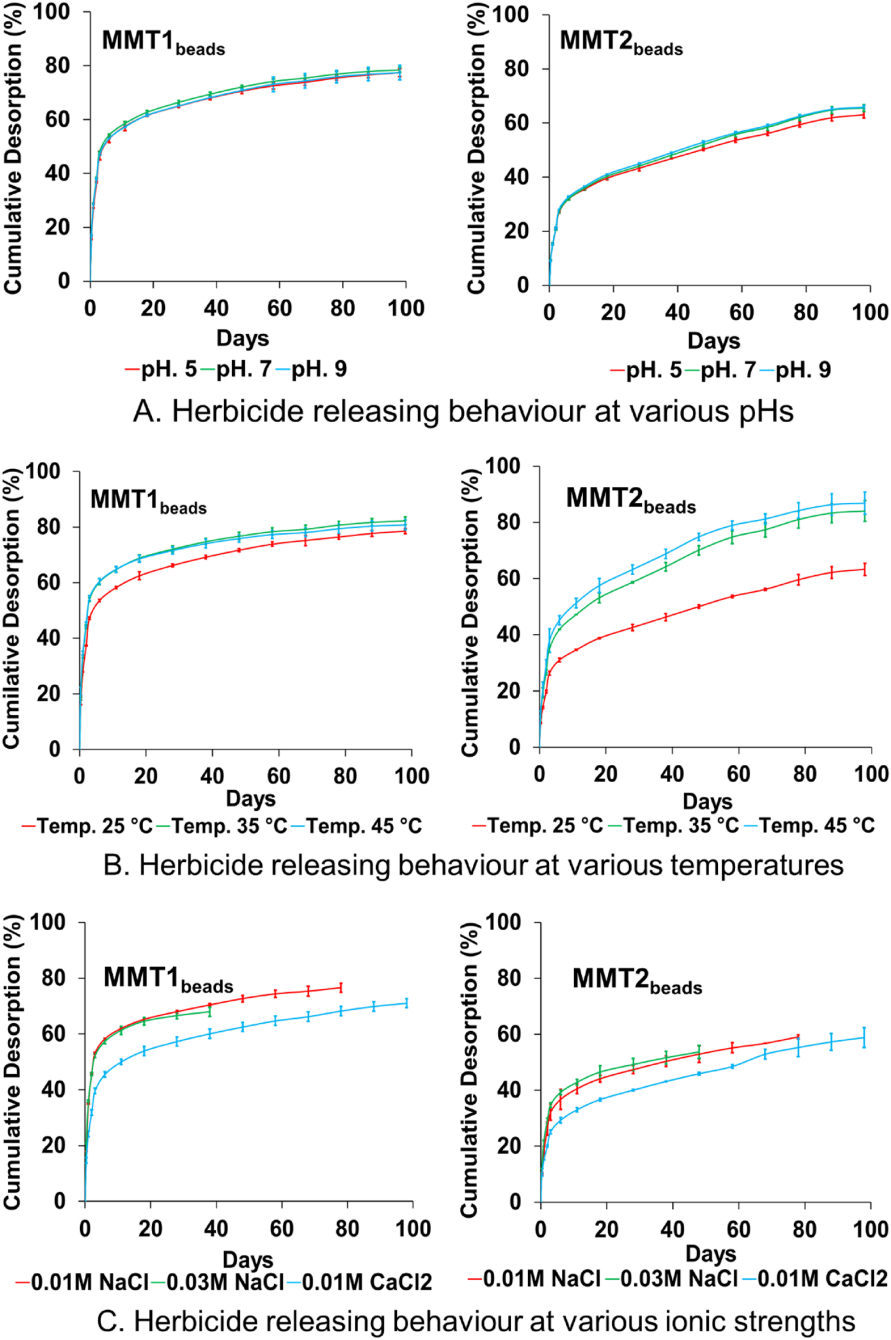
Cumulative desorption percentage of 2,4-D from synthesised microbeads at (**A**) various pH, (**B**) various temperatures and (**C**) various ionic strengths in lab conditions.

Results revealed that both synthesised beads (MMT1_Beads_ and MMT2_Beads_) have excellent herbicide releasing behaviour at various pHs and temperatures (Fig. 6A,B respectively). There was no evidence of significant variations in cumulative desorption percentages for either microbeads, except temperature variations for MMT2_Beads_.

The herbicide-releasing behaviour of MMT1_Beads_ was faster than MMT2_Beads_ at the initial stage, whereas MMT2_Beads_ showed a sharply increasing rate throughout the experiments. However, both beads showed long-term (~ 100 days) herbicide-releasing behaviour at a wide range of pHs (5–9) and temperatures (25–45 °C). The variations in herbicide-releasing behaviour for MMT2_Beads_ at various temperatures could be attributed to the silane group of the surfactants used for clay modification having strong binding capacity with 2–4-D in the presence of sodium alginate, hindering the release of 2–4-D at low temperatures.

To investigate the influences of ionic strengths and index cation, we ran the experiments using NaCl solutions at 0.01 M and 0.03 M and CaCl_2_ solutions at 0.01 M (ionic strength 0.01 M CaCl_2_ ≅ 0.03 M NaCl). Results revealed that herbicide-releasing behaviour of both microbeads is greatly influenced by various ionic strengths as well as index cation (Fig. 6C). The monovalent Na^+^ ions can easily interact with sodium-alginate, resulting softened the microbeads and diluted into water with increasing time and NaCl concentration. Two different concentrations (0.01 M to 0.03 M) of NaCl were used, and beads were diluted quicker when NaCl concentration was higher (0.03 M). After certain period, when the beads were diluted into water, then all active ingredients came out from the beads and diluted into water. We did not use those samples for further investigations. The MMT2_Beads_ showed slightly stronger protective capacity against dilution at higher NaCl concentrations. This is due to the presence of APTES on the organoclays, as APTES have a strong binding capacity to the broken edge of the clay minerals (Waddell et al., 1981, Su et al., 2012). The data were recorded until the microbeads were not fully diluted in the presence of NaCl. However, both microbeads retained their original granular structures and had good herbicide-releasing behaviour in 0.01 M CaCl_2_ solutions, meaning that both microbeads perform better in calcareous than sodic soils.

The two different surfactants-modified organoclays showed distinctly different 2–4-D adsorption capacity (mg g^−1^) prior to the synthesis of microbeads. This was due to the different active sites of the organoclays and the different interaction mechanisms between each organoclay and 2,4-D. Therefore, the synthesised microbeads (MMT1_Beads_ and MMT2_Beads_) followed distinct desorption patterns as well (Supplementary Tables 3 and Supplementary Table 4). Pore water samples collected from the pots at various time intervals were analysed and results confirmed the presence of 2,4-D until 57 and 48 days after application for MMT1_Beads_ and MMT2_Beads_, respectively (Supplementary Table 5). This result confirms that the synthesised microbeads will have slow-and long-term herbicide-releasing behaviour. Previous published reports confirm our findings, where slow and long term herbicide release were achieved using different carriers and coating materials^41,42^. Our investigation further revealed that pH has significant effect on 2,4-D releasing behaviour, and similar results were reported by^43^. The herbicide-releasing speed of MMT2_Beads_ was slightly faster than MMT1_Beads_ to begin with, whereas MMT1_Beads_ showed a sharply increasing rate throughout the experiments. Therefore, fast- or slow-releasing formulations can be synthesised using the carrier materials MMT2 and MMT1, respectively. In addition, release speed can be customised using different proportions of MMT1 and MMT2 during microbead synthesis. This paper enhance our understanding of the interaction mechanisms between herbicide active ingredients and carrier materials for synthesising effective CFRs, that will have less toxicity compared with conventional formulations. Sodium alginate were used for bead synthesis, which is a biodegradable natural polymers, and have no toxicological effect on soil and environment, rather it will improve soil health and structure^44–46^.

### Weed control efficacy of synthesised microbeads

The results revealed that both microbeads have excellent weed control efficacy (WCE) at various dosages for different weed species (Table 4). The expected WCE (> 80%) was achieved at different dosages and different days after herbicide application for various weed species. MMT1_Beads_ require a smaller amount and less time than MMT2_Beads_ to control the three different weed species, suggesting that the herbicide-releasing capacity of MMT2_Beads_ is slower than MMT1_Beads_. Nevertheless, both (MMT1_Beads_ and MMT2_Beads_) have excellent WCE on the target pest.

**Table 4:** Weed control efficacy of synthesized microbeads.

Herbicide application rate Pot^−1^ (mg)	Sowthistle (*Sonchus oleraceus*)			Fleabane (*Conyza bonariensis*)			Pigweed (*Chenopodium album*)		
	No. of weeds Pot^−1^	Dry weight (mg)	WCE (%)	No. of weeds Pot^−1^	Dry weight (mg)	WCE (%)	No. of weeds Pot^−1^	Dry weight (mg)	WCE (%)
MMT1_Beads_									
1000	22 ± 1	2144.8 ± 49.6	73.93 ± 0.92	22 ± 2	353.5 ± 13.7	88.49 ± 0.26	8 ± 1	695.4 ± 29.8	67.22 ± 1.78
2000	23 ± 3	1415.7 ± 19.5	82.79 ± 0.03	24 ± 1	318.4 ± 11.3	89.63 ± 0.20	9 ± 1	587.5 ± 11.15	72.31 ± 0.21
3000	26 ± 1	1159.8 ± 34.1	85.90 ± 0.58	20 ± 3	238.2 ± 3.1	92.24 ± 0.03	9 ± 2	456.6 ± 5.18	78.47 ± 0.49
5000	24 ± 3	824.6 ± 28.3	89.98 ± 0.46	–		–	7 ± 1	386.8 ± 12.23	81.76 ± 0.37
MMT2_Beads_									
1000	22 ± 1	2831.4 ± 121.1	65.59 ± 1.06	24 ± 2	563.6 ± 16.2	81.65 ± 0.23	9 ± 1	1258.2 ± 28.93	40.69 ± 2.05
2000	21 ± 4	2387.6 ± 60.9	70.98 ± 1.09	20 ± 1	498.2 ± 23.4	83.78 ± 0.50	8 ± 1	1025.2 ± 38.34	51.67 ± 1.25
3000	23 ± 1	1962.5 ± 15.3	76.15 ± 0.47	23 ± 2	353.4 ± 16.69	88.49 ± 0.73	11 ± 1	854.4 ± 32.67	59.72 ± 2.01
5000	19 ± 2	1204.7 ± 121.1	85.36 ± 1.30	–	–	–	9 ± 1	504.2 ± 11.43	76.23 ± 0.26
CF	25 ± 1	855.4 ± 12.2	89.60 ± 0.27	23 ± 2	394.2 ± 21.46	87.16 ± 0.49	10 ± 1	365.7 ± 7.78	82.76 ± 0.57
Control	24 ± 1	8227.9 ± 98.5	–	19 ± 3	3071.5 ± 50.0	–	13 ± 1	2121.2 ± 24.5	–
LSD_(0.05)_	NS	218.1495	2.5412	5.9787	65.9023	1.1592	NS	72.91443	3.9578

CF = Commercial Formulation; Control = Untreated.

2,4-D is a systemic herbicide and the synthesised microbeads were applied to the soil in the pot experiment. The microbeads slowly released herbicide AIs and plant root-tips absorbed them from the soil, transporting them throughout the plant through xylem tissues, slowly killing the plant. This is in contrast to traditional foliar spraying of commercial herbicide formulations, where the herbicide AIs are absorbed through the plant leaf stomata, then transported throughout the plant through xylem and phloem tissues, thereby also killing the plants.

## Conclusion

Various sorption kinetic models were applied to the experimental data and results revealed that adsorption of 2,4-D onto both organo-montmorillonites follows the PSO kinetic model rather than PFO and is predominately controlled by the chemisorption process as the rate-limiting step. Both organoclays follow the Elovich model, which infers they have energetically heterogeneous surfaces. The intra-particle diffusion is not the sole rate-limiting step for the entire adsorption process however, as multi-step mechanisms were involved in adsorption of 2,4-D onto both organoclays. Desorption studies deploying various experimental conditions revealed that both organoclays interact strongly with 2,4-D and showed excellent desorption behaviour at a wide range of pH (5–9), temperatures (25–45 °C), and ionic strengths. The monovalent Na^+^ ions easily interact with sodium-alginate, causing the beads to soften and become diluted more rapidly than when exposed to CaCl_2_ solutions. This suggests that both MMT1_Beads_ and MMT2_Beads_ will have far greater weed control efficacy in calcareous than sodic soils. However, both MMT1_Beads_ and MMT2_Beads_ revealed excellent broad-leaf weed control efficacy under glasshouse conditions.

Both organoclays proved to be suitable carrier materials for synthesising microbeads for CRFs of anionic herbicides like 2,4-D. The herbicide-releasing speed of MMT1_Beads_ was faster than MMT2_Beads_ to begin with, whereas MMT2_Beads_ showed a sharply increasing rate throughout the experiments. Fast- or slow-releasing formulations can therefore be synthesised using the carrier materials MMT1 and MMT2, respectively. Furthermore, release can be controlled using different proportions of MMT1 and MMT2 during microbead synthesis.

### Supplementary Information

Supplementary Information 1.

Supplementary Information 2.

## Data Availability

All data generated or analysed during this study are included in its supplementary information files.
