# Supplementary material for: Controlled release herbicide formulation for effective weed control efficacy
Source: Sci Rep. 2024 Feb 20;14:4216. doi: 10.1038/s41598-024-53820-8 (PMC10879510; doi:10.1038/s41598-024-53820-8)
Supplement: Supplementary file 1 — Supplementary Information 1. [file 41598_2024_53820_MOESM1_ESM.docx]

Controlled release herbicide formulation for effective weed control efficacy

**Santosh Kumar Paul^a,b,c^, Yunfei Xi^d^, Peter Sanderson^a,b^, Ravi Naidu^a,b*^**

**^a^** Global Centre for Environmental Remediation (GCER), ATC Building, The University of Newcastle, Callaghan, NSW 2308, Australia

**^b^** CRC for Contamination Assessment and Remediation of the Environment (CRC CARE), ATC Building, The University of Newcastle, Callaghan, NSW 2308, Australia

**^c^** Agronomy Division, Bangladesh Agricultural Research Institute (BARI), Joydebpur, Gazipur 1701, Bangladesh

**^d^** Central Analytical Research Facility (CARF) & School of Chemistry and Physics - Faculty of Science, Queensland University of Technology, Brisbane, QLD 4001, Australia

***Corresponding author**

**Laureate Professor Ravi Naidu**

Address: Global Centre for Environmental Remediation, The University of Newcastle, University Dr, Callaghan, NSW 2308, Australia.

Contact: Email: [Ravi.Naidu@newcastle.edu.au](mailto:Ravi.Naidu@newcastle.edu.au) (R. Naidu); T: +61249138705

# **Materials and Reagents**

Two organoclays were purchased from Sigma-Aldrich, Australia and denoted as MMT1 and MMT2 for ODA and ODA+APTES-modified montmorillonites, respectively. These organoclays were used without further purification. As per information provided by the supplier, some properties of these organoclays are presented (Supplementary Table 1). The model herbicide 2,4-D (PESTANAL^®^, analytical standard, purity > 98%) was also purchased from Sigma-Aldrich, Australia and its physicochemical properties are presented in Supplementary Table 2.

Table S1. Physicochemical properties of organoclays

| Specification | Properties | | | Remarks |
| --- | --- | --- | --- | --- |
|  |  | MMT1 | MMT2 |  |
| Commercial name |  | Nanomer® I.30 E | Nanomer® I.31PS | As per supplier |
| Surface modification |  | 25 wt.% octadecylamine | 0.5-5 wt.% aminopropyltriethoxysilane  15-35 wt.% octadecylamine | As per supplier |
| Colour |  | Off-White to Beige | Off white | As per supplier |
| Appearance |  | Powder | Powder | As per supplier |
| Weight loss in drying |  | ≤ 3% | ≤ 3% | As per supplier |
| Molecular weight |  | 125.69 g mol^-1^ | 294.19 g mol^-1^ | As per supplier |
| Size |  | ≤ 20 μm | ≤ 20 μm | As per supplier |
| Bulk density |  | 200-500 kg m^-3^ | 200-500 kg m^-3^ | As per supplier |
| Organic carbon content |  | 25.305% | 22.942% | Determined |
| pH in water |  | 7.62 (1% w/v) | 4.26 (1% w/v) | Determined |

Table S2. Physicochemical properties of 2,4-D

| Parameters | Properties |  | Parameters | Properties |
| --- | --- | --- | --- | --- |
| IUPAC name | 2,4-Dichlorophenoxyacetic acid |  | Melting point | 140.5 ^o^C |
| Synonyms | 2,4-D |  | Boiling point | 160 ^o^C |
| Chemical formula | C_8_H_6_Cl_2_O_3_ |  | Density | 1.57 g cm^-3^ |
| Molecular weight | 221.04 g mol^-1^ |  | log Kow | 2.81 |
| Classification | Organic compound, Phenoxy herbicide, Glycolates |  | [IDLH (Immediate danger)](https://www.google.com/search?q=2,4-dichlorophenoxyacetic+acid+idlh+immediate+danger&sa=X&ved=2ahUKEwijiamO2ePwAhV1yzgGHUFrB-kQ6BMoADAlegQIIxAC) | 100 mg m^-3^ |
| Appearance | White to off-white |  | Dissociation constants | pKa1 = -4.9  pKa2 = 2.81 |
| Odour | Odourless |  | Koc | 20-136 |
| UV-Vis sensitivity | 229 nm and 282 nm |  | Vapour pressure | 1.4 x 10^-7^ mmHg |
| Hydrogen bond donor site | 1 |  | Henry's constant | 8.6 x 10^-6^ atm.m^3^ mol^-1^ |
| Hydrogen bond acceptor site | 3 |  | Herbicide group | Phenoxy acetic acid group |
| Solubility | In water = 900 mg L^-1^  In acetone =168.25 mg L^-1^  In ethanol =103 mg L^-1^ |  | Half-life/Stability | 7 days (soil)  15 days (aerobic aquatic)   - 1. ys (anaerobic aquatic) |

# **Preparation of a UV-Vis spectrophotometry standard curve for 2,4-D analysis**

To measure the concentration of 2,4-D through UV-Vis spectrophotometry, a standard curve was prepared. For this context, 2,4-D solutions of different concentrations (2, 4, 8, 12, 16, 20, 24, 32, 40, 48 mgL^-1^) were prepared. Then the absorbance was measured using a UV-Vis spectrophotometer (Model: UV-3600 Plus, UV-VIS-NIR Spectrophotometer, SHIMADZU, Japan) at λ_max_ of 229 nm. The absorbance values at different concentrations were plotted on a graph following absorbance versus concentration (Supplementary Fig. 1). Then a UV-Vis spectrophotometry standard curve was prepared and the equation obtained from the correlation curve helped to determine unknown 2,4-D concentration.


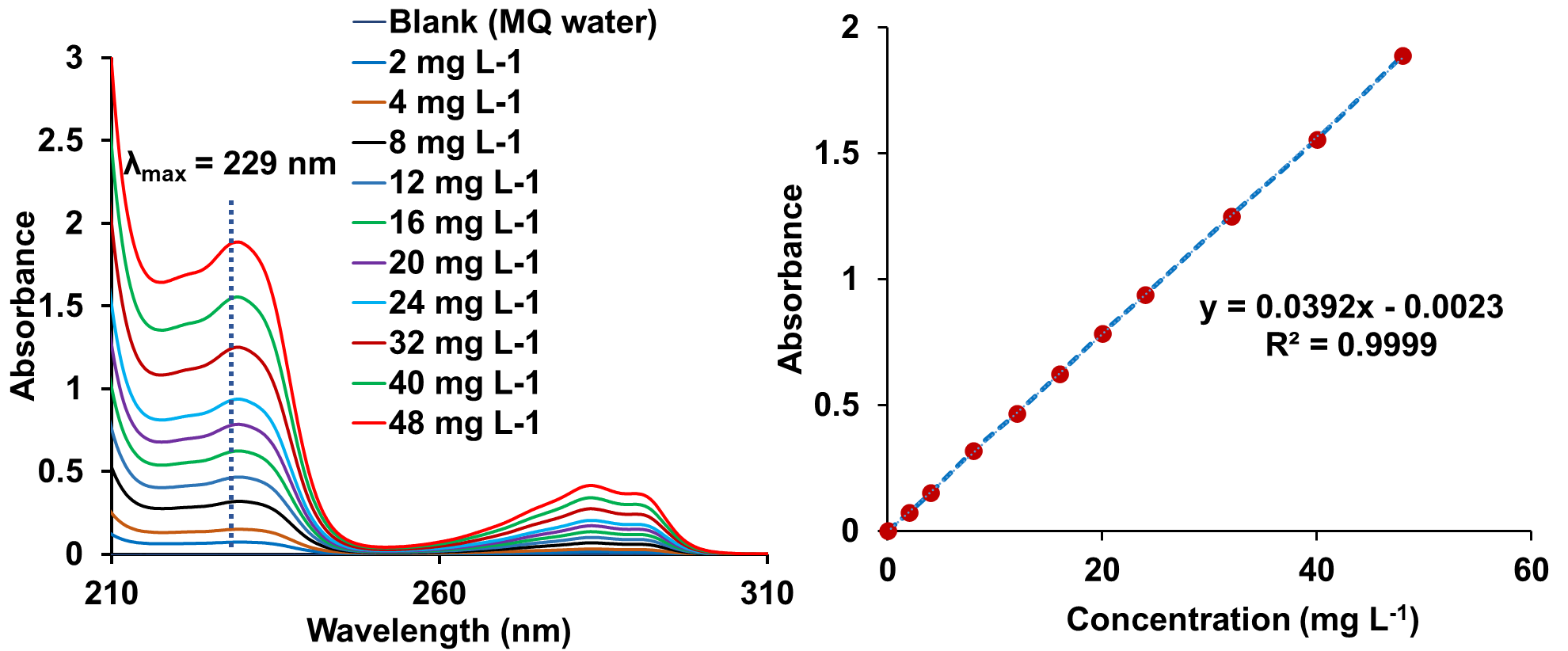


Fig. S1. UV–Vis spectrum of different 2,4-D solutions (Left) and a calibration curve (Right).

# **Herbicide sorption kinetics**

For CRFs of herbicide, it is expected that the AIs adsorbed onto the carriers during the adsorption process would be released in a desired way at a certain concentration that can control the target pest and maintain the pest population below the toxicological threshold level. In our previous experiments, it emerged that adsorption was the highest at acidic conditions (pH.3) for both organoclays [1]. Therefore, adsorption kinetics studies were investigated at pH.3. For sorption kinetic studies, 25 mg of each organoclay (MMT1 or MMT2) was added into a 50 ml centrifuge tube containing 20 ml 2,4-D solution (100 mgL^-1^) at pH = 3.03 and the tubes were shaken for a specific time using an orbital shaker (RATEK, Model: OM6, Australia). For each adsorbent, the total number of centrifuge tubes were 12 and equilibration periods were 1, 3, 6, 9, 12, 15, 20, 30, 45, 60, 90, and 120 seconds. After shaking for a predetermined time, the suspensions were centrifuged at 4500 rpm (model; Sorvall LYNX 4000 Centrifuge, Thermo Scientific, Germany) for 15 minutes at 25 ^°^C and filtered using 0.25µm cellulose acetate filters. 2,4-D concentrations in the supernatants were determined using a UV-VIS spectrophotometer (Model: UV-3600 Plus, UV-VIS-NIR Spectrophotometer, SHIMADZU, Japan) at λ_max_ of 229 nm. After adsorption, using the standard curve from Fig. S1, the remaining concentrations of 2,4-D in the solutions were determined. The adsorbed amounts of 2,4-D per gram of adsorbents Q_t_ (mg g^-1^) values were determined following the equation (Eq. S1) and plotted as time (min) versus Q_t_ (mg g^-1^) (Supplementary Fig. 2). The figure reveals that adsorption is very rapid at the initial stage and reaches a state of equilibrium within the first 20 minutes for both organoclays. After 20 minutes no significant changes in the adsorption of 2,4-D onto the adsorbents occurred. However, complete adsorption studies were carried out for 120 minutes for both organoclays. The adsorption was replicated twice, and the mean values are presented.

Sorption capacities at specific time Q_t_ (mg g^-1^) and equilibrium condition Q_e_ (mg g^-1^), expressed as quantity of adsorbate (herbicide) adsorbed by per unit mass of adsorbent (organoclays), were calculated by the equations below, respectively:

$$Q_{t}=\frac{{(C}_{0}-C_{t})V}{M} S1$$

$$Q_{e}=\frac{{(C}_{0}-C_{e})V}{M} S2$$

where C_0_, C_t_ and C_e_ are denoted as herbicide concentrations (mg L^-1^) at initial, at a specific time (t), and at equilibrium condition, respectively. V (L) and M (g) represent the volume of herbicide solution and mass of the organoclay.


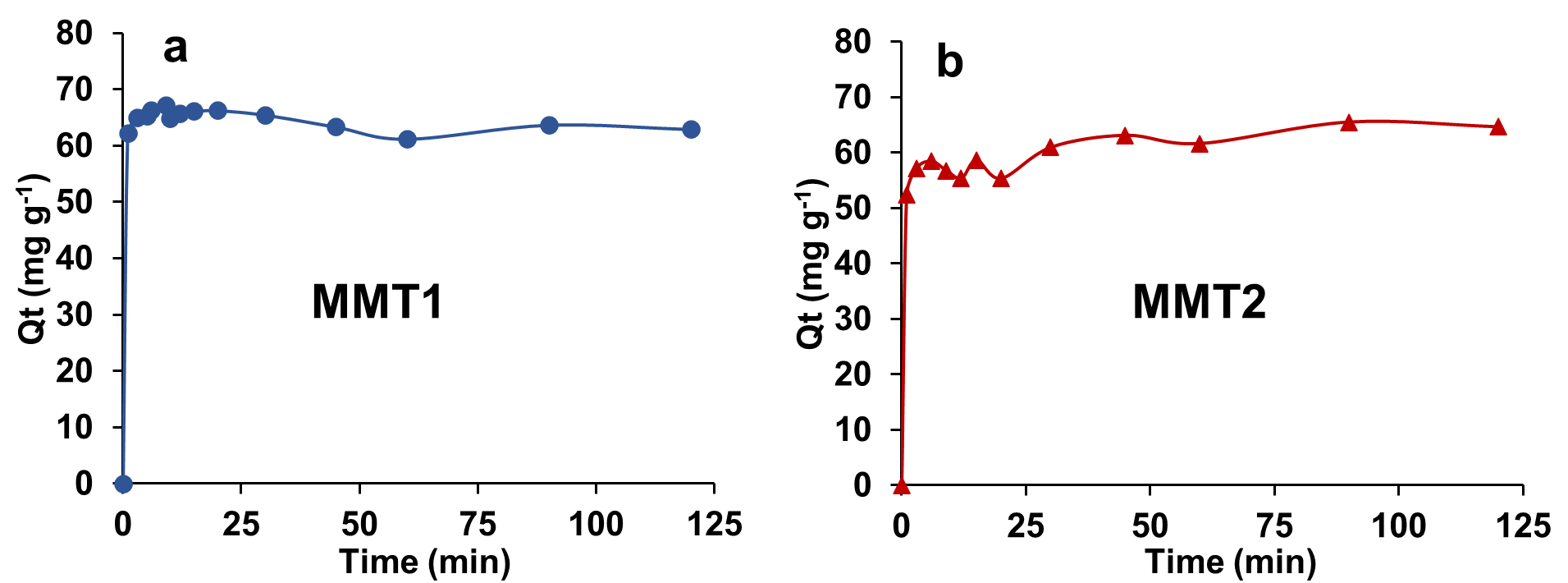


Fig. S2. Equilibrium adsorption studies of 2,4-D to determine the adsorption time for (a) MMT1 and (b) MMT2

Further adsorption experiments were conducted using same experimental conditions like kinetics experiment, and various kinetics models including pseudo-first-order (PFO), pseudo-second-order (PSO), Elovich and intra-particle diffusion models were applied to explore the experimental data.

Pseudo-first-order, otherwise widely known as the Lagergren kinetics model, was firstly described by Lagergren in 1898, while the linear form of the pseudo-first-order equation was devised later [2-4] and expressed as:

$$\mathrm{Ln}\left( Q_{e}-Q_{t} \right)= Ln Q_{e}- k_{1}t S3$$

Where k_1_ denotes Lagergren’s first-order rate constant (min^-1^) calculated from the slope and intercept of the graph plotted as ln(Q_e_-Q_t_) versus t. The pseudo-second-order kinetics model describes the chemisorption process of the adsorbates to the adsorbents [3, 5, 6]. This model can be expressed as an equation below, where k_2_ represents the pseudo-second-order rate constant (g mg^-1^ min^-1^). Q_t_, Q_e_ and t are similar as that mentioned earlier:

$$\frac{t}{Q_{t}}= \frac{1}{k_{2}{Q_{e}}^{2}} +\frac{t}{Q_{e}} S4$$

The Elovich kinetic model describes the surface heterogeneity of the adsorbents which means multilayer sorption to adsorbents [7]. It is well recognised as being able to describe the chemisorption process when adsorption takes place on the heterogeneous adsorptive sites [5, 8, 9]. The model is very applicable when sorption occurs following a wide range of activation energy and the surface of the adsorbents are energetically heterogeneous [7, 10, 11]. This model can describe the PSO kinetics without involved interaction mechanisms between solutes and sorbents [12-14]. Meanwhile the Elovich model suggests the two-step adsorption process incorporates the following: (i) rapid adsorption at the initial stage on the readily available external adsorptive sites; and (ii) slow adsorption onto the outer and inner pores of the sorbents through diffusion [11, 12, 15]. The model is expressed as:

$$Q_{t}= \frac{1}{\beta}\ln\left( \alpha\beta\right)+\frac{1}{\beta}\ln\left( t \right) S5$$

Where t and Q_t_ have a similar meaning as mentioned earlier where α (mg g^-1^ min^-1^) is the initial sorption kinetic and β (g mg^-1^) is the sorption constant related to the extent of surface coverage activation energy for chemisorption [5, 8, 9]. The constants α and β are calculated from the slope of the plotting as Q_t_ versus lnt. Weber Jr and Morris [9] proposed an intra-particle diffusion model and suggested that intra-particle diffusion is the rate-controlling factor and varies with square root of time. The multi-step adsorption process can be well described using the intra-particle diffusion model. In any adsorption process, if intra-particle diffusion is the only interaction mechanism and sole rate-controlling step, then the plot of t^1/2^ versus Q_t_ must be linear with no intercept and passing through the origin (C=0) [8, 16, 17]. If the curve does not pass through the origin (C ≠ 0), then it is suggested that intra-particle diffusion is not the sole rate-limiting step for the entire adsorption process.

The intercept value of the plot represents film thickness of the boundary layer and indicates the extent of boundary layer effect [18, 19]. The higher intercept value indicates greater boundary layer effect to the adsorption process [19]. Moreover, if the plot shows non-linearity, then it could be assumed that more than one step is controlling the entire adsorption process and more complex interaction mechanisms were involved simultaneously during adsorption [8, 16, 17]. The model can be expressed as written in equation 6 below, where k_p_ is the intra-particle diffusion rate constant (mg g^-1^ min^-1/2^) and C is the intercept which gives an impression of film thickness of the boundary layer [19]. k_p_ and C are calculated from the slope and intercept of linear plot of Q_t_ versus t^1/2^. It is suggested that the sorption process is controlled by only intra-particle diffusion if the linear plot of Q_t_ versus t^1/2^ passes through the origin, i.e. C=0 [8], and sorption could be controlled by more than one mechanism when the plot is multi-linear [18].

$$Q_{t}= k_{p}t^{1/2}+C S6$$

A two-constant rate kinetics model was used to fit the experimental data. The model can be expressed in a following linear equation [20, 21].

$$\text{Ln(}C_{0}-C_{t})=\text{a}+bln t S7$$

Where C_0_ and C_t_ are similar to that mentioned earlier, whereas a is the initial herbicide adsorption rate constant and b is the adsorption rate coefficient of the model. Values of a and b are calculated using the slope and intercept of the plot ln (C_0_-C_t_) versus lnt.

# **Soil sampling and characterisation**

Experimental soils were collected from Fletcher, NSW, Australia (**latitude 32°52'36"S and longitude 151°38'22"E)** for desorption studies in a pot experiment under glasshouse conditions. Five different spots were randomly selected, and soils from each spot were collected from surface horizons (0–15 cm) and mixed properly to form a composite soil sample. Collected soils were air-dried for 24 hours, and passed through a 2-mm diameter sieve, then stored in hermetic bottles at room temperature (25 ± 1 ◦C) until required for use. Surface area (SA) of the selected soil was analysed by nitrogen adsorption and desorption isotherms using a Micromeritics Tristar II 3020 SA and porosity analyser (USA). Prior to SA analysis, the samples were finely powdered and degassed at 110 °C for 12 hours using Micromeritics VacPrep 061 Sample Degas System (USA). 5g of soils were suspended in 25 ml ultrapure Milli-Q water, thereafter electrical conductivity (EC) and pH were recorded using Laqua, manufactured by Horiba Scientific.

Cation exchange capacity (CEC) was determined using BaCl_2_ compulsive exchange method [22, 23]. Soil textural class was determined through the hydrometer method based on sand, silt, and clay percentage [24]. Total organic carbon (TOC), nitrogen (N), and sulphur (S) were determined following the methods described in Chapter 3. For elemental compositions, 5 ml aqua regia were added to 0.5 g soil samples and digested through the Microwave Digestion System (MARS 6 240/50, USA). The supernatant was filtered using a 0.45 µm cellulose acetate (CA) filter and major elements were determined using inductively coupled plasma optical emission spectrometer (ICP-OES; Avio^TM^ 200, PerkinElmer^®^, United States). The minerals in the soil were identified from peaks of x-ray diffraction (XRD) analysis using PANalytical X’pert Pro diffractometer fitted with Cu-Kα radiation (λ = 1.54 Å) and generated voltage of 45 kV with an applied current of 40 mA. The data were recorded over a range of 5–80° 2θ with a step size of 0.0263° at the scanning rate of 1° 2θ min^−1^. Some physicochemical properties of the selected soil are presented in Table 1.

# **Results and discussions**

Table S3. Herbicide desorption capacity of MMT1_Beads_

| Active ingredient desorbed from synthesized microbeads at various experimental conditions (mg/g) | | | | | | | | | | | | | | | | |  |  |  |
| --- | --- | --- | --- | --- | --- | --- | --- | --- | --- | --- | --- | --- | --- | --- | --- | --- | --- | --- | --- |
| Days | |  | pH | | | | |  | Temperatures | | | | |  | Ionic strengths and index cation | | | | |
|  | |  | pH.5 |  | pH.7 |  | pH.9 |  | 25 °C |  | 35 °C |  | 45 °C |  | 0.01M NaCl |  | 0.03M NaCl |  | 0.01M CaCl2 |
| 0 | |  | 0.00±0.00 |  | 0.00±0.00 |  | 0.00±0.00 |  | 0.00±0.00 |  | 0.00±0.00 |  | 0.00±0.00 |  | 0.00±0.00 |  | 0.00±0.00 |  | 0.00±0.00 |
| 0.333 | |  | 15.22±0.02 |  | 16.52±0.01 |  | 16.42±0.02 |  | 15.70±0.00 |  | 17.77±0.05 |  | 20.33±0.14 |  | 17.44±0.01 |  | 20.23±0.01 |  | 14.30±0.01 |
| 1 | |  | 26.41±0.01 |  | 28.21±0.00 |  | 27.79±0.01 |  | 27.11±0.01 |  | 32.51±0.05 |  | 33.68±0.07 |  | 34.04±0.02 |  | 35.01±0.04 |  | 23.50±0.02 |
| 2 | |  | 35.75±0.02 |  | 37.41±0.00 |  | 36.93±0.02 |  | 36.31±0.01 |  | 42.89±0.07 |  | 43.72±0.08 |  | 44.64±0.02 |  | 44.38±0.05 |  | 30.97±0.00 |
| 3 | |  | 45.30±0.16 |  | 46.76±0.01 |  | 45.78±0.02 |  | 45.87±0.04 |  | 52.43±0.07 |  | 52.93±0.09 |  | 51.68±0.02 |  | 50.77±0.07 |  | 38.49±0.00 |
| 6 | |  | 51.46±0.15 |  | 52.78±0.04 |  | 51.58±0.01 |  | 52.07±0.04 |  | 58.62±0.11 |  | 58.80±0.10 |  | 56.62±0.02 |  | 55.72±0.08 |  | 44.25±0.00 |
| 11 | |  | 55.91±0.16 |  | 56.98±0.07 |  | 55.75±0.03 |  | 56.60±0.04 |  | 62.93±0.10 |  | 63.01±0.11 |  | 60.26±0.03 |  | 59.65±0.10 |  | 48.65±0.01 |
| 18 | |  | 59.89±0.04 |  | 61.00±0.07 |  | 59.93±0.03 |  | 60.71±0.04 |  | 66.92±0.12 |  | 66.64±0.12 |  | 63.43±0.05 |  | 62.78±0.10 |  | 52.46±0.63 |
| 28 | |  | 63.24±0.05 |  | 64.58±0.07 |  | 63.34±0.02 |  | 64.34±0.05 |  | 70.08±0.11 |  | 69.60±0.13 |  | 66.10±0.05 |  | 64.73±0.09 |  | 55.74±0.63 |
| 38 | |  | 66.20±0.07 |  | 67.54±0.07 |  | 66.35±0.02 |  | 67.24±0.06 |  | 72.69±0.11 |  | 72.00±0.16 |  | 68.45±0.05 |  | 66.08±0.10 |  | 58.44±0.65 |
| 48 | |  | 68.67±0.10 |  | 70.11±0.06 |  | 68.93±0.04 |  | 69.68±0.06 |  | 74.62±0.13 |  | 73.78±0.13 |  | 70.66±0.07 |  | - |  | 60.79±0.66 |
| 58 | |  | 70.57±0.13 |  | 72.15±0.08 |  | 71.13±0.27 |  | 71.80±0.08 |  | 76.21±0.13 |  | 75.16±0.14 |  | 72.30±0.07 |  | - |  | 62.94±0.67 |
| 68 | |  | 71.84±0.12 |  | 73.34±0.09 |  | 72.37±0.27 |  | 73.09±0.08 |  | 77.04±0.14 |  | 75.89±0.15 |  | 73.23±0.08 |  | - |  | 64.40±0.66 |
| 78 | |  | 73.40±0.09 |  | 74.77±0.10 |  | 73.83±0.23 |  | 74.30±0.08 |  | 78.50±0.13 |  | 77.20±0.16 |  | 74.47±0.06 |  | - |  | 66.37±0.68 |
| 88 | |  | 74.51±0.11 |  | 75.72±0.09 |  | 74.74±0.25 |  | 75.54±0.10 |  | 79.47±0.13 |  | 78.13±0.18 |  | - |  | - |  | 67.97±0.67 |
| 98 | |  | 75.29±0.15 |  | 76.27±0.09 |  | 75.30±0.27 |  | 76.35±0.09 |  | 79.98±0.14 |  | 78.52±0.18 |  | - |  | - |  | 69.07±0.66 |
| LSD_(0.05_) | |  | 0.3160 |  | 0.2011 |  | 0.4567 |  | 0.1808 |  | 0.3332 |  | 0.4051 |  | 0.1370 |  | 0.1801 |  | 1.5351 |

Table S4. Herbicide desorption capacity of MMT2_Beads_

| Active ingredients desorbed from synthesized microbeads at various experimental conditions (mg/g) | | | | | | | | | | | | | | | | | | |  |  |
| --- | --- | --- | --- | --- | --- | --- | --- | --- | --- | --- | --- | --- | --- | --- | --- | --- | --- | --- | --- | --- |
| Days | |  | pH | | | | |  | Temperatures | | | | |  | Ionic strengths and index cation | | | | | |
|  | |  | pH.5 |  | pH.7 |  | pH.9 |  | 25°C |  | 35°C |  | 45°C |  | 0.01M NaCl |  | 0.03M NaCl |  | 0.01M CaCl2 | |
| 0 | |  | 0.00±0.00 |  | 0.00±0.00 |  | 0.00±0.00 |  | 0.00±0.00 |  | 0.00±0.00 |  | 0.00±0.00 |  | 0.0±0.00 |  | 0.00±0.00 |  | 0.00±0.00 | |
| 0.333 | |  | 6.74±0.00 |  | 6.73±0.02 |  | 6.51±0.01 |  | 6.30±0.02 |  | 7.84±0.03 |  | 8.76±0.09 |  | 7.38±0.04 |  | 8.66±0.01 |  | 7.03±0.04 | |
| 1 | |  | 10.95±0.02 |  | 11.08±0.04 |  | 10.97±0.02 |  | 10.27±0.04 |  | 13.99±0.17 |  | 14.93±0.25 |  | 13.34±0.15 |  | 15.95±0.01 |  | 11.00±0.00 | |
| 2 | |  | 15.00±0.03 |  | 15.16±0.05 |  | 15.25±0.02 |  | 14.32±0.04 |  | 19.26±0.08 |  | 20.85±0.23 |  | 18.98±0.24 |  | 21.02±0.10 |  | 14.46±0.04 | |
| 3 | |  | 19.59±0.04 |  | 19.72±0.05 |  | 20.27±0.00 |  | 18.99±0.07 |  | 25.22±0.12 |  | 27.40±0.41 |  | 23.18±0.28 |  | 25.08±0.08 |  | 18.16±0.07 | |
| 6 | |  | 23.12±0.04 |  | 23.14±0.06 |  | 23.64±0.02 |  | 22.35±0.07 |  | 30.23±0.01 |  | 32.61±0.16 |  | 26.52±0.35 |  | 28.33±0.08 |  | 21.14±0.10 | |
| 11 | |  | 25.71±0.04 |  | 25.96±0.07 |  | 26.45±0.01 |  | 24.97±0.02 |  | 34.03±0.00 |  | 37.03±0.17 |  | 29.30±0.18 |  | 30.96±0.11 |  | 23.86±0.07 | |
| 18 | |  | 28.61±0.07 |  | 29.10±0.09 |  | 29.66±0.01 |  | 28.00±0.02 |  | 38.37±0.18 |  | 41.52±0.25 |  | 31.88±0.12 |  | 33.58±0.23 |  | 26.50±0.05 | |
| 28 | |  | 31.28±0.10 |  | 31.86±0.07 |  | 32.50±0.02 |  | 30.74±0.11 |  | 42.36±0.02 |  | 45.64±0.16 |  | 34.23±0.15 |  | 35.55±0.22 |  | 28.92±0.02 | |
| 38 | |  | 33.81±0.01 |  | 34.73±0.10 |  | 35.41±0.02 |  | 33.41±0.13 |  | 46.35±0.15 |  | 4967±0.16 |  | 36.39±0.19 |  | 37.30±0.22 |  | 31.17±0.01 | |
| 48 | |  | 36.31±0.04 |  | 37.55±0.08 |  | 38.25±0.04 |  | 36.12±0.06 |  | 50.61±0.17 |  | 54.05±0.13 |  | 38.24±0.30 |  | 38.79±0.23 |  | 33.17±0.05 | |
| 58 | |  | 38.72±0.07 |  | 40.28±0.09 |  | 40.72±0.04 |  | 38.71±0.04 |  | 53.95±0.22 |  | 56.98±0.16 |  | 39.84±0.18 |  | - |  | 35.03±0.06 | |
| 68 | |  | 40.54±0.07 |  | 42.16±0.10 |  | 42.66±0.04 |  | 40.51±0.04 |  | 55.86±0.26 |  | 58.64±0.18 |  | 41.02±0.00 |  | - |  | 38.21±0.17 | |
| 78 | |  | 42.89±0.09 |  | 44.81±0.11 |  | 45.17±0.05 |  | 43.00±0.19 |  | 58.46±0.30 |  | 60.80±0.28 |  | 42.69±0.07 |  | - |  | 39.93±0.33 | |
| 88 | |  | 44.72±0.11 |  | 46.78±0.13 |  | 47.00±0.05 |  | 44.47±0.21 |  | 60.13±0.34 |  | 62.31±0.38 |  | - |  | - |  | 41.37±0.30 | |
| 98 | |  | 45.50±0.12 |  | 47.33±0.13 |  | 47.56±0.05 |  | 45.69±0.22 |  | 60.64±0.37 |  | 62.68±0.39 |  | - |  | - |  | 42.49±0.36 | |
| LSD_(0.05)_ | |  | 0.2014 |  | 0.2565 |  | 0.0913 |  | 0.3299 |  | 0.5951 |  | 0.7412 |  | 0.5511 |  | 0.3751 |  | 0.4812 | |

Table S5. Concentration of 2,4-D in collected soil pore water

| Days of soil pore water collection (DAA) |  | 2,4-D concentration in collected soil pore water (mg/L) | | |
| --- | --- | --- | --- | --- |
|  |  | MMT1_Beads_ |  | MMT1_Beads_ |
| 2 |  | 84.67±0.12 |  | 53.98±0.47 |
| 9 |  | 60.55±0.18 |  | 51.65±0.58 |
| 23 |  | 42.76±0.41 |  | 51.13±0.76 |
| 30 |  | 37.17±0.06 |  | 48.57±0.41 |
| 37 |  | 34.85±0.06 |  | 26.59±0.41 |
| 41 |  | 30.31±0.06 |  | 22.52±0.06 |
| 45 |  | 24.21±0.23 |  | 21.71±0.06 |
| 48 |  | 22.41±0.18 |  | 10.55±0.06 |
| 53 |  | 21.71±0.06 |  | - |
| 57 |  | 10.31±0.06 |  | - |
| LSD_(0.05)_ |  | 0.9820 |  | 1.3953 |

DAA = Days After Application

# **References**

1. Paul, S.K., et al., Investigation of herbicide sorption-desorption using pristine and organoclays to explore the potential carriers for controlled release formulation. *Chemosphere*, 2023. **337**: p. 139335.

2. Azizian, S., Kinetic models of sorption: a theoretical analysis. J*. Col. .Inter. Sci*., 2004. **276**(1): p. 47-52.

3. Ho, Y.-S., Review of second-order models for adsorption systems. *J. Hazard. Mater.*, 2006. **136**(3): p. 681-689.

4. Yuh-Shan, H., Citation review of Lagergren kinetic rate equation on adsorption reactions. *Scientometrics*, 2004. **59**(1): p. 171-177.

5. Ho, Y.-S. and G. McKay, Pseudo-second order model for sorption processes. Process Biochemistry, 1999. **34**(5): p. 451-465.

6. Salvestrini, S., et al., Modelling the biphasic sorption of simazine, imidacloprid, and boscalid in water/soil systems. *J. Environ. Sci. .Health*, Part B, 2014. **49**(8): p. 578-590.

7. Zeldowitsch, J., Adsorption site energy distribution. *Acta PhysPhysicochim, URSS*, 1934. **1**(1): p. 961-973.

8. Chen, S., et al., Removal of Cr (VI) from aqueous solution using modified corn stalks: Characteristic, equilibrium, kinetic and thermodynamic study. *Chem. Eng. J*., 2011. **168**(2): p. 909-917.

9. Weber Jr, W.J. and J.C. Morris, Kinetics of adsorption on carbon from solution. *J. Sanit. Eng. Div*., 1963. **89**(2): p. 31-59.

10. Rudzinski, W. and T. Panczyk, Kinetics of isothermal adsorption on energetically heterogeneous solid surfaces: a new theoretical description based on the statistical rate theory of interfacial transport. *J. Phy. Chem. B.*, 2000. **104**(39): p. 9149-9162.

11. Aharoni, C. and F. Tompkins, Kinetics of adsorption and desorption and the Elovich equation, in Advances in Catalysis. 1970, , Vol. 21 (pp. 1-49). Elsevier. p. 1-49.

12. Wu, F.-C., R.-L. Tseng, and R.-S. Juang, Characteristics of Elovich equation used for the analysis of adsorption kinetics in dye-chitosan systems. Chem. Eng. J., 2009. **150**(2-3): p. 366-373.

13. Sparks, D.L., Environmental soil chemistry. 2003: 2^nd^ ed. Elsevier.

14. Sparks, D.L., Kinetics of soil chemical processes. 2013: Academic Press.

15. Cáceres, L., et al., Modeling the sorption kinetic of metsulfuron-methyl on Andisols and Ultisols volcanic ash-derived soils: Kinetics parameters and solute transport mechanisms. *J. Hazard. Mater.*, 2010. **179**(1-3): p. 795-803.

16. Báez, M.E., E. Fuentes, and J. Espinoza, Characterization of the atrazine sorption process on andisol and ultisol volcanic ash-derived soils: kinetic parameters and the contribution of humic fractions. *J. Agri. Food Chem*., 2013. **61**(26): p. 6150-6160.

17. Han, R., et al., Characterization of modified wheat straw, kinetic and equilibrium study about copper ion and methylene blue adsorption in batch mode. *Carbohydrate Poly*., 2010. **79**(4): p. 1140-1149.

18. Chio, C.-P., M.-C. Lin, and C.-M. Liao, Low-cost farmed shrimp shells could remove arsenic from solutions kinetically. *J. Hazard. Mater*., 2009. **171**(1-3): p. 859-864.

19. Kannan, N. and M.M. Sundaram, Kinetics and mechanism of removal of methylene blue by adsorption on various carbons—a comparative study. *Dyes and Pigments*, 2001. **51**(1): p. 25-40.

20. Alghanmi, S.I., et al., Acid leaching of heavy metals from contaminated soil collected from Jeddah, Saudi Arabia: kinetic and thermodynamics studies. *Int. Soil and Water Cons. Res*., 2015. **3**(3): p. 196-208.

21. Aliyu, M.K., et al. In Situ Remediation of Lead Contaminated Marine Sediment using Bentonite, Kaolin and Sand as Capping Materials. in IOP Conference Series: Earth and Environ. Sci.. 2020. IOP Publishing.

22. Hendershot, W.H. and M. Duquette, A simple barium chloride method for determining cation exchange capacity and exchangeable cations. Soil Sci. Soci. Amer. J., 1986. **50**(3): p. 605-608.

23. Horn, D., M. Alley, and P. Bertsch, Cation exchange capacity measurements. Com. Soil Sci. Plant Ana., 1982. **13**(10): p. 851-862.

24. Gee, G.W. and D. Or, 2.4 Particle‐size analysis. Methods of soil analysis: Part 4 physical methods, 2002. **5**: p. 255-293.
